# PET Molecular Imaging: Old Habits Do Not Die, They Only Evolve into New Applications

**DOI:** 10.3390/ijms25010403

**Published:** 2023-12-28

**Authors:** Ferdinando F. Calabria

**Affiliations:** Department of Nuclear Medicine and Theranostics, Mariano Santo Hospital, 87100 Cosenza, Italy; f.calabria@aocs.it; Tel.: +39-328-3337036

The first studies on human applications of radioisotopes for the in vivo targeting of pathophysiological processes began in the late 1930s in Western Europe and the USA with ^99m^Tc [1,2], which is currently the most widely used tracer in traditional nuclear medicine. A further γ-emitting agent, ^131^I, enabled imaging and treatment of the thyroid tissue [3]; although this is not strictly speaking a radiopharmaceutical, its affinity for nonradioactive iodine has enabled the visualization and treatment of hyperthyroidism since the early days of nuclear medicine imaging. It is highly and selectively absorbed in the target tissue [3].

Undoubtedly, the advent of theranostic in the last decade has its roots in the preliminary, pioneering noninvasive treatment of hyperthyroidism with ^131^I. A huge volume of data reports that the synergy between diagnostic tracers in nuclear medicine and their therapeutic counterpart is enlarging the application of theranostic in prostate cancer cases with radiolabeled Prostate Specific Membrane Antigen (PSMA) [4] and neuroendocrine tumors with ^68^Ga/^177^Lu-labeled somatostatin analogs [5]. Moreover, upcoming studies are evaluating the role of ^177^Lu in the treatment of severe meningiomatosis [6].

This rapid process of innovation would not have taken place without the development and commercial availability of hybrid scanners such as Single Photon Emission Computed Tomography (SPECT) [7] and Positron Emission Tomography (PET) [8], combined with Computed Tomography (CT). In fact, both SPECT/CT and PET/CT improve the localization of functional data to accurately identify and monitor the diseases.

Its increased power resolution has made it possible to consider PET imaging among the main diagnostic procedures in oncology, cardiology, and neurology, thanks to the advantages of ^18^F-FDG [9] as an analog of glucose. However, ^18^F-FDG presents limits in evaluating the cortex, the myocardium, and malignant tumors, with a low rate of glucose metabolism. Several tracers have earned a special place in the field of PET molecular imaging. The noteworthy experience of nuclear physicians in imaging neuroendocrine tumors with somatostatin analogs and SPECT has been copied and improved by PET [10]. Additionally,^11^C/^18^F radiolabeled choline has gained a role in the management of prostate cancer patients [11] and ^87^Rb has been used for myocardial perfusion imaging [12], while amino acid and amyloid tracers currently enable the study of brain tumors [13] and dementia [14], respectively.

Interestingly, the identification of diagnostic pitfalls linked to the uptake of radiolabeled choline in several benign tumors has enlarged its field of application to the diagnosis of parathyroid adenoma [15]. This is an intuitive example of the vitality of nuclear medicine, where a prostate cancer imaging agent can play a further diagnostic role due to the overexpression of phosphatidylcholine turnover in parathyroid adenoma.

At the same time, a performing radiopharmaceutical, ^18^F/^68^Ga-PSMA, is quickly becoming the most important agent for the imaging of prostate cancer and is considered as a preliminary step prior to submitting metastatic patients to radionuclide therapy with ^177^Lu [16].

Nevertheless, beyond the incidental discovery of new diagnostic applications, the development of novel PET radiopharmaceuticals is a complex, scheduled process, involving a number scientists worldwide. Radiopharmacists, physicists, biologists, and chemical engineers are some professional groups which are actively involved in this continuous growth and innovation process, alongside nuclear medicine researchers.

The intention of this Special Issue is to expand the knowledge regarding PET radiopharmaceuticals that are currently under study or to describe new applications of tracers already in use. The expected benefit is to share current topics of interest in order to connect molecular and preclinical studies with clinical works.

As a matter of fact, the interest in the PET imaging with ^125^I and ^131^I is still high; their optimal half-life enables their availability in PET centers not provided by a cyclotron, while novel synthesis strategies enable high lipophilic iodine-containing compounds, which may improve their pharmacological and pharmacokinetic properties. Their selective accumulation in the target tissues (i.e., thyroid and stomach) enables one to conceive a feasible role of therapy with radioiodine-containing drugs in several diseases [17].

The potential impact of consolidated tracers in the management of cancer patients should not be underestimated; interesting data are emerging from the application of radiomics to PET imaging, with ^18^F-fluoroethylcholine in prostate cancer imaging [18] or ^18^F-DOPA in movement disorders [19]. The other contributions of this Special Issue offer direct insight into a wide range of new molecular pathways—such as hypoxia imaging and the ^68^Ga-Fibroblast-Activation Protein Inhibitor—novel hybrid chelators, and new synthesis methods through an experimental approach; however, there is still a lack of knowledge on the potential benefits and pitfalls emerging from routine use of the proposed tracers, which should be assessed in different clinical settings.

The availability of the latest hybrid imaging systems, such as PET/CT and PET/Magnetic Resonance Imaging (MRI), is probably the primary research direction for recognizing patterns of physiological bio-distribution and the investigation of their diagnostic accuracy, regarding the creation of a wide field of applications concerning radiopharmaceutical preparations for PET imaging and therapy, as well as multimodal imaging [8,20].

In conclusion, it is likely that soon the body of knowledge surrounding these topics will increase, eventually defining the role of theranostic in the context of personalized medicine. This needs to be achieved through a bridge between all professionals involved.

It is unlikely that new tracers will replace ^18^F-FDG. Conversely, it is quite exciting to imagine that these tracers could play a significant role in the future of nuclear medicine, including earlier theranostic applications. Among tested tracers, the most effective theranostic agent for personalized imaging and treatment of diseases will be found.
